# Case Report: Diverse clinical phenotypes and treatment responses in pediatric autoimmune nodopathy: a case series

**DOI:** 10.3389/fimmu.2026.1978921

**Published:** 2026-10-30

**Authors:** Ya Guo, Jie Song, Chao Zhang, Na Chen, Jiamin Guo

**Affiliations:** Department of Pediatric, Shandong Provincial Hospital Affiliated to Shandong First Medical University, Jinan, Shandong, China

**Keywords:** autoimmune nodopathy, case report, clinical phenotypes, pediatric, rituximab

## Abstract

**Background:**

Autoimmune nodopathy (AN) is a recently identified category of immune-mediated peripheral neuropathy defined by circulating auto-antibodies against nodal and paranodal cell-adhesion molecules.

**Methods:**

We retrospectively reviewed three pediatric patients with antibody-positive AN diagnosed by cell-based assay (CBA).

**Results:**

Three male patients aged 5 years 7 months to 13 years 8 months were positive for anti-NF186 (1:32), anti-Caspr1 (1:10), and anti-NF155 (1:100) IgG, respectively. The anti-NF186-positive patient and the anti-Caspr1-positive patient presented with a CIDP/GBS-like phenotype, whereas the anti-NF155-positive patient presented with tremor, sensory ataxia, and limb weakness resembling the adult AN phenotype. These patients also had markedly elevated CSF protein and electrophysiological findings of reduced or unobtainable CMAP and SNAP amplitudes; in addition, the NF155-positive patient showed slowed motor conduction velocity. All patients received first-line therapy with IVIG and intravenous methylprednisolone pulses followed by an oral prednisone taper. Two patients responded to this regimen, and the NF155-positive patient was refractory to first-line therapy but improved after rituximab.

**Conclusion:**

In this small series, pediatric AN is heterogeneous, and antibody subtyping with clinical assessment is essential for diagnosis. First-line immunotherapy was effective in NF186- and Caspr1-positive patients, whereas the NF155-positive patient improved only after adding rituximab. Early recognition, prompt diagnosis, and timely therapeutic intervention are of paramount importance for improving the prognosis of pediatric AN.

## 1 Introduction

Autoimmune nodopathy (AN) is a recently identified category of immune-mediated peripheral neuropathy defined by circulating auto-antibodies against nodal and paranodal cell-adhesion molecules that are essential for saltatory conduction (1). Formally distinguished from chronic inflammatory demyelinating polyradiculoneuropathy (CIDP) in the 2021 EAN/PNS diagnostic guidelines, this entity marks a paradigm shift from histopathological classification toward antibody-defined nosology (2). Although AN and CIDP overlap in clinical manifestations, electrophysiological findings, and cerebrospinal fluid examination, they can still be differentiated by their distinct features, especially in terms of pathology, therapeutic outcomes, and prognosis. It is estimated to account for 5-10% of patients meeting the diagnostic criteria for CIDP, although this proportion is gradually increasing with growing recognition of the condition (1). Patients with AN commonly present with acute or subacute, frequently severe, sensorimotor neuropathy characterized by disproportionately prominent sensory ataxia, action tremor, or cranial nerve involvement, and exhibit poor response to intravenous immunoglobulin (IVIG) which constitutes the first-line therapy for CIDP (3). At present, the main therapeutic strategies for AN encompass three categories: B-cell depletion and modulation, reduction of IgG levels, and complement inhibition (4).

The pathogenic mechanisms of AN have yet to be fully elucidated, however, current evidence implicates a complex interplay involving antibody-producing cells, immunoglobulin sub-classes, epitope localization, and antibody - epitope binding (3). To date, the following antibodies targeting cell adhesion molecules at the axoglial junctions of the node of Ranvier in peripheral nerves have been identified as being associated with AN: neurofascin-155(NF155), contactin-1 (CNTN1), contactin-associated protein 1 (Caspr1), and nodal neurofascin or pan-neurofascin (pan-NF) (3). These antibodies are predominantly of the IgG4 subclass, which possesses the following characteristics: (1) inability to fix complement; (2) failure to bind or upregulate the inhibitory FcγRIIB receptor; (3) incapacity to recruit immune cells via Fc receptors, thereby failing to induce phagocytosis or trigger cytokine release and inflammatory responses; and (4) resistance to neutralization by anti-idiotypic antibodies, these properties may explain why AN patients respond poorly to IVIG (5–7). The clinical manifestations of AN vary depending on the specific auto-antibody involved, and the antibody subclass of a given auto-antibody may differ across different disease stages in the same patient (8). Pathological findings show variable axonal loss with minimal or no myelin destruction, indicating that AN is neither a demyelinating nor an inflammatory disease (5).

In this study, we aimed to delineate the clinical characteristics of three pediatric patients with antibody-positive AN. Increasing recognition of AN as a new disease notwithstanding, the literature is heavily weighted toward adult patients, and data on children remain sparse. Our findings add to the growing body of pediatric AN data and may offer guidance for refining diagnostic and therapeutic strategies in this population, thereby helping to fill a considerable void in the current literature.

## 2 Methods

Peripheral blood (3 mL) was collected from each subject, and serum antibodies were detected using a cell-based assay at Hangzhou Dian Medical Laboratory Center. The full-length sequences of NF155, CNTN1, NF186, CNTN2, and Caspr1 were cloned into the pcDNA3.1 vector to construct recombinant plasmids. HEK293T cells were cultured in DMEM supplemented with 15% fetal bovine serum and 1% penicillin–streptomycin, seeded onto culture dishes, and transiently transfected with the plasmids using polyethylenimine (PEI) transfection reagent. At 48h after transfection, the cells were fixed with pre-cooled acetone at 4°C for 10min and stored after drying. The transfected and fixed cells incubated with serum (diluted 1:10 to 1:1000) for 1 h and washed three times with phosphate-buffered saline (PBS), the slides were incubated with FITC-conjugated anti-human IgG secondary antibody (1:200) for 30min at room temperature. After three additional PBS washes, immunoreactivity was visualized under a fluorescence microscope.

## 3 Case reports

### 3.1 Case 1

A 9-year-10-month-old male presented with a 4-month history of progressive, worsening weakness in both lower limbs, accompanied by distal extremity paresthesia. Neurological examination revealed bilateral absence of patellar and Achilles tendon reflexes. Cerebrospinal fluid (CSF) analysis showed elevated protein levels (0.85 g/L) without pleocytosis, indicating albuminocytologic dissociation. Motor conduction studies suggested partial conduction block in the evoked compound muscle action potential (CMAP) amplitudes of the bilateral tibial nerves, and sensory conduction studies suggested reduced distal sensory nerve action potential (SNAP) amplitudes in the bilateral ulnar nerves and the left superficial peroneal nerve. These findings are suggestive of a mixed demyelinating and axonal sensorimotor polyneuropathy. Serum testing was positive for anti-neurofascin-186 (NF186) IgG antibodies at a titer of 1:32, while magnetic resonance imaging (MRI) of the lumbosacral plexus and nerve roots revealed no significant abnormalities. The patient was treated with a 5-day course of IVIG (2g/kg) followed by intravenous methylprednisolone pulse therapy (20 mg/kg/day for 3 days), and then transitioned to a tapering regimen of oral prednisone. At discharge, the patient exhibited substantial motor improvement and was able to walk independently. At the 4-month follow-up, his neurological function had fully normalized, NCS showed partial improvement, and serum anti-NF186 IgG antibody had seroconverted to negative. Oral corticosteroid tapering is ongoing.

### 3.2 Case 2

A 5-year-7-month-old boy presented with a 20-day history of progressively worsening, ascending weakness involving all four limbs, accompanied by limb pain. Neurological examination revealed bilaterally diminished patellar and Achilles tendon reflexes. CSF analysis showed a markedly elevated protein level of 2.39 g/L, consistent with albuminocytologic dissociation. Nerve conduction studies demonstrated reduced CMAP amplitudes with slowed motor conduction velocities (MCV) in the bilateral median, ulnar, tibial, and common peroneal nerves, while distal SNAPs were unobtainable. Serum testing was positive for anti-Caspr1 IgG antibody at a titer of 1:10. The patient was treated with a 3-day course of IVIG (2g/kg total) combined with two courses of intravenous methylprednisolone pulse therapy (20mg/kg/day for 3 days each), followed by a tapering regimen of oral prednisone. His weakness improved markedly after treatment; he regained the ability to stand independently and walk with assistance. At the 1-month follow-up, serum anti-Caspr1 IgG antibodies had seroconverted to negative. By the 18-month follow-up, his neurological function had fully normalized. Oral corticosteroid therapy was continued for 2 years before being gradually tapered and subsequently discontinued, and then NCS had essentially returned to baseline levels.

### 3.3 Case 3

A 13-year-8-month-old boy was referred for evaluation of a 2-month history of postural abnormality and tremor, which were followed over the subsequent 20 days by progressively worsening lower limb weakness and limb numbness. Neurological examination revealed decreased tactile and pain sensation, bilaterally absent patellar and Achilles tendon reflexes, and positive finger-to-nose test and Romberg sign, suggesting cerebellar or proprioceptive dysfunction. CSF analysis demonstrated a markedly elevated protein level of 5.91 g/L. Electrophysiological studies revealed prolonged distal motor latencies in the bilateral median, ulnar, tibial, and common peroneal nerves, with CMAP amplitudes that were either reduced or unobtainable and partially slowed MCV; distal SNAPs were bilaterally unelicitable. Needle electromyography of the bilateral gastrocnemius muscles showed scattered denervation potentials with reduced recruitment on maximal effort. Serum testing was positive for anti-NF155 IgG antibody at a titer of 1:100, and MRI of the lumbosacral plexus and nerve roots revealed nerve root thickening with segmental linear enhancement. Then the patient received IVIG (2g/kg over 5 days) and intravenous methylprednisolone pulse therapy (15 mg/kg/day for 3 days), followed by an oral prednisone taper. However, his lower limb weakness showed only mild improvement, and he continued to experience tremor and plantar numbness. At the 6-month follow-up, repeat antibody testing revealed a decline in titer to 1:10; nevertheless, his tremor, lower limb weakness, and plantar numbness remained largely unchanged, without significant clinical improvement. Given this incomplete response to first-line immunotherapy, rituximab was initiated at 9 months from disease onset, administered at a dose of 375 mg/m²weekly for 4 consecutive weeks. Following rituximab therapy, peripheral B-cell counts decreased to zero, and concomitant corticosteroids were administered and gradually tapered. Approximately 3 months after rituximab therapy (at the 12-month follow-up), the patient showed clinical improvement, characterized by reduced tremor, increased lower-limb muscle strength, and regained ability to ascend and descend stairs independently. Repeat testing at that time revealed an anti-NF155 antibody titer of 1:10. At the 14-month follow-up, only partial improvement was observed in his NCS.

## 4 Discussion

In this case series, we describe three pediatric patients with antibody-defined AN, targeting NF186 (n=1), Caspr1 (n=1), and NF155 (n=1)(**Figure 1**). Our cases illustrate the variability of presentations that can be seen in pediatric AN, although this heterogeneity should not be over-interpreted given the small number of patients per antibody subtype. As illustrated in Cases 1 and 2, the patients positive for anti-NF186 and anti-Caspr1 antibodies, respectively, exhibited a classic CIDP/GBS-like phenotype characterized by symmetric or ascending limb weakness, hyporeflexia, and albuminocytologic dissociation, without prominent tremor or ataxia (9–11). Case 3, a patient seropositive for anti-NF155 antibodies, exhibited the prototypical adult AN phenotype, characterized by subacute tremor, sensory ataxia (positive finger-to-nose test), distal numbness, marked motor slowing with unelicitable sensory potentials, and MRI evidence of nerve root thickening with enhancement (12). This combination of tremor and disproportionate sensory impairment in a demyelinating neuropathy is a recognized hallmark of NF155-AN, attributed to paranodal disruption and secondary myelin loosening. Our cases are consistent with the concept that the nodal or paranodal localization of target antigens shapes the pattern of physiological dysfunction, and underscore the importance of antibody subtyping, interpreted together with the clinical picture, when AN is suspected. These diverse presentations emphasize the need for a high index of suspicion for AN in children with unexplained sensory, motor, or even sensory-dominant symptoms, particularly when accompanied by CSF protein elevation or electrophysiological abnormalities. In atypical or low-titer cases, confirmatory assays such as ELISA and teased-fiber immunohistochemistry are advisable.

**Figure 1:**
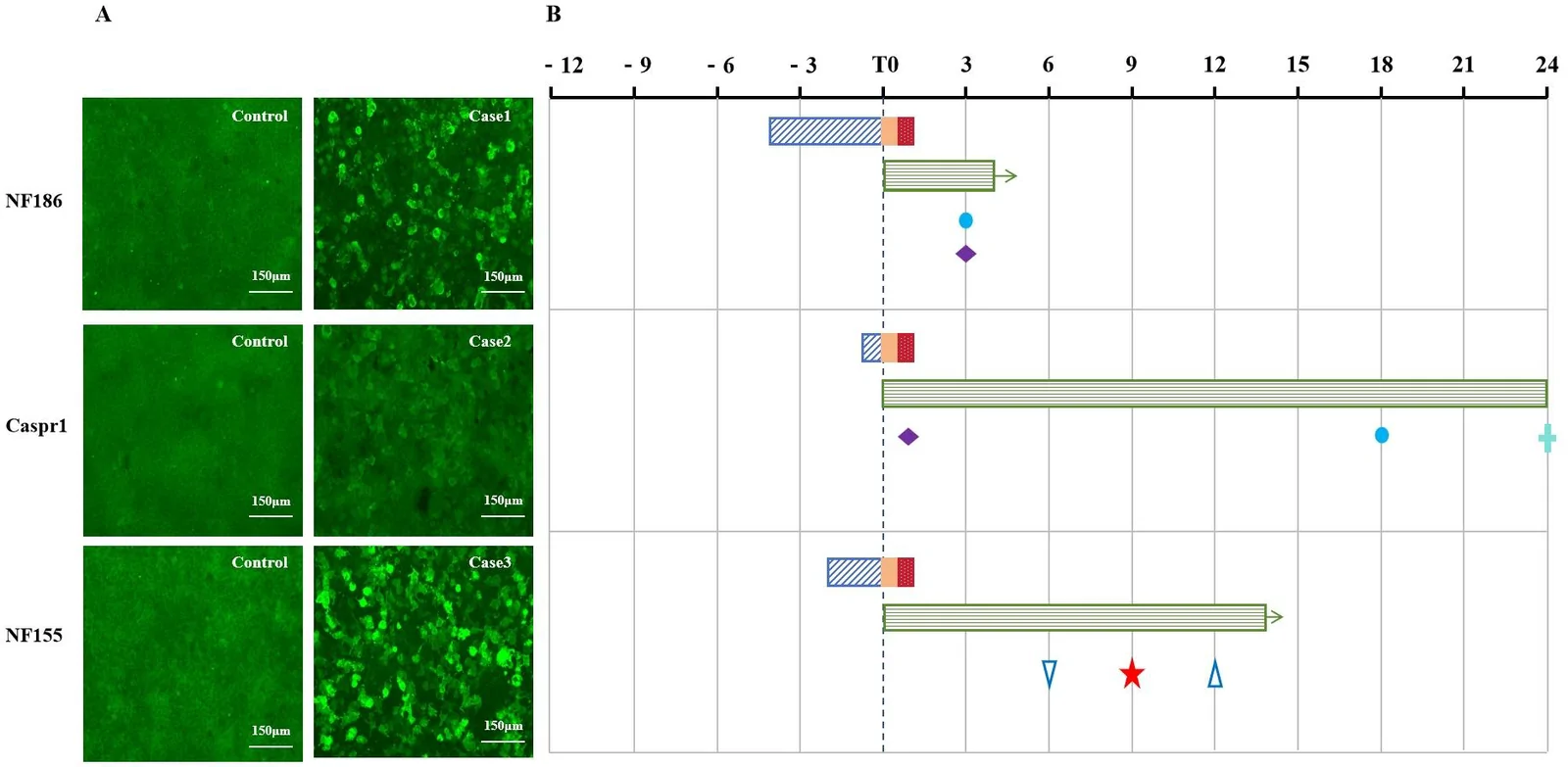
Immunofluorescence findings and clinical treatment timelines of four cases with AN. **(A)** Representative immunofluorescence images from four patients. Cell-based assay (CBA) showing positive immunofluorescence staining of patient serum on HEK293 cells transfected with the indicated target antigens: neurofascin-186 (NF186; Cases 1), contactin-associated protein 1 (Caspr1; Case 2), and neurofascin-155 (NF155; Case 3). **(B)** Clinical courses and treatment timelines for each patient are aligned to disease onset/diagnosis (T0, dashed vertical line), with months on the horizontal axis. AN, autoimmune nodopathy; IVIG, intravenous immunoglobulin; IVMP, intravenous methylprednisolone. Scale bar=150μm.

The published data on pediatric AN are limited to isolated case reports and small case series. Accordingly, the comparisons presented below are descriptive and warrant cautious interpretation. Anti-NF155: Pediatric anti-NF155 disease has been reported to present with distal weakness and sensory symptoms; a recent single-center series of six children from Beijing further described male predominance and a chronic course (13). Early pediatric reports described severe CIDP phenotypes, characterized by partial responses to corticosteroids and favorable responses to B-cell depletion among IgG4-positive cases (14). Our NF155-positive patient presented with tremor and sensory ataxia, showed a partial response to IVIG and steroids, and benefited from rituximab. This presentation resembles the adult phenotype, but whether it is typical of children remains uncertain.Anti-NF186/pan-neurofascin: In a pediatric series of four patients with anti-neurofascin antibodies, IgG2-predominant antibodies were detected in three, who demonstrated variable but generally favorable responses to IVIG (15). This finding is consistent with the favorable first-line responses observed in our NF186-positive patient; however, antibody subclass typing was not performed. Anti-Caspr1/CNTN1: Pediatric contactin-1 disease has been reported only in isolated cases, including a CIDP-like case (16) and a case with membranous nephropathy (17). We identified no pediatric anti-Caspr1 series; therefore, our Case 2 cannot be meaningfully compared at the subtype level. In adults, antibodies against the contactin-1/Caspr1 complex are frequently IgG4 and are associated with poor response to IVIG (18), which contrasts with the favorable response in our patient; however, this discrepancy may reflect case selection or antibody subclass rather than age.

The literature illustrated that the pathogenicity of nodoparanodal antibodies is shaped not only by the antigenic target, but also by both subclass and valency (4). The majority of autoantibodies implicated in AN are of the IgG4 subclass, and the underlying pathogenic mechanisms have been detailed in the Introduction section. Other IgG subclasses capable of complement fixation have also been described, particularly in cases with acute-onset presentations, which may partially account for the clinical heterogeneity observed across different antibody subtypes (19, 20). However, due to the retrospective nature of this case series, IgG subclass identification was not performed in our pediatric patients, which precludes further subclass-specific interpretation. Accumulating evidence indicates that antibody titers track with clinical status, and both titers and serum neurofilament light chain (sNfL) correlate with disease severity at nadir. Accordingly, both have been suggested as biomarkers for follow-up and therapeutic decision-making (3, 5). In Cases 1 and 2, seroconversion to antibody negativity closely paralleled clinical remission, consistent with previous reports linking antibody titers to clinical status; however, given that only two patients were involved, these observations are insufficient to establish the utility of antibody titers as a biomarker. In Case 3, however, a substantial decline in anti-NF155 titer at six months was not accompanied by corresponding clinical improvement, and significant functional recovery only occurred following the addition of rituximab. This discordance between antibody titer and clinical improvement in NF155-associated disease suggests that once structural paranodal damage is established, a more profound reduction in pathogenic antibodies-achieved through B-cell depletion-or a longer recovery period may be required. It also cautions against using titer decline alone as a surrogate marker for treatment adequacy in this antibody subgroup. This dissociation highlights the importance of early diagnosis and aggressive intervention to prevent long-term disability.

Current therapeutic strategies for AN fall into three main categories: B-cell depletion and modulation, IgG reduction, and complement inhibition (4, 21). In our case series, two patients (the NF186-positive case and the Caspr1-positive case) showed clinical improvement after first-line immunotherapy, whereas the NF155-positive patient improved only after the addition of rituximab; this finding is consistent with previous reports (22). Given the heterogeneity in treatment regimens and follow-up durations, these observations remain descriptive and cannot be formally compared with adult data. This differential responsiveness may be attributable to the following factors: (1) at least in pediatric patients with NF186- or Caspr1-associated AN, first-line immunotherapy may yield better outcomes than adult literature would suggest; and (2) antibody subclass and associated pathogenic mechanisms—although the absence of IgG subclass analysis in this study precludes confirmation of which subclasses were involved. By contrast, the treatment-refractory course in the NF155-positive patient aligns with the well-established link between anti-NF155 antibodies and the IgG4 subclass documented in adult populations (23). These observations are consistent with the notion that rituximab represents a reasonable second-line therapeutic option for pediatric patients who respond inadequately to first-line therapy, particularly those with NF155-positive, likely IgG4-mediated disease; nevertheless, this view warrants confirmation in larger studies.

Given that the existing literature on AN is derived overwhelmingly from adult cohorts, these cases contribute meaningfully to the sparse pediatric data and highlight the heterogeneity of clinical presentations, antibody profiles, therapeutic responses, and long-term outcomes in this underrecognized population. Nevertheless, several limitations of this study should be acknowledged. First, the generalizability of our findings is constrained by the small cohort size and the single-center design. As each antibody subtype was represented by only one patient, subtype-specific comparisons with other pediatric reports are descriptive in nature and cannot support inferences regarding differences in clinical features or treatment response. And standardized longitudinal outcomes supporting these comparisons were not recorded. Second, the absence of IgG subclass typing precludes definitive validation of the mechanistic hypotheses proposed. Third, the considerable variability in follow-up duration across cases, along with the lack of standardized corticosteroid tapering protocols, may have confounded the observed interpatient differences in therapeutic outcomes. Finally, neither ELISA nor teased-nerve immunohistochemistry was performed for independent confirmation, and no patient in this pediatric cohort underwent nerve biopsy; therefore, we could not directly correlate antibody specificities with underlying histopathological changes.

## 5 Conclusion

We report three children with antibody-positive AN targeting NF186, Caspr1, and NF155, whose presentations ranged from a CIDP/GBS-like phenotype to the prototypical adult AN phenotype of tremor and sensory ataxia. The anti-NF186- and anti-Caspr1-positive patients responded well to IVIG and corticosteroids, whereas the anti-NF155-positive patient improved only after rituximab. This heterogeneity supports a high index of suspicion for AN in children with unexplained neuropathy, particularly with CSF protein elevation, and underscores the value of antibody testing. Given the small sample, single-center design, and absence of IgG subclass typing, these findings are descriptive and warrant validation in larger pediatric cohorts. Nevertheless, these observations suggest a potential subtype-specific treatment pattern and support the conduct of prospective, multicenter studies with standardized regimens and longitudinal follow-up. Clinicians should place particular emphasis on antibody testing in children presenting with neuropathy of unclear etiology or atypical sensory manifestations. Early recognition, prompt diagnosis, and timely therapeutic intervention are of paramount importance for improving the prognosis of pediatric AN.

## Data Availability

The original contributions presented in the study are included in the article/Supplementary Material. Further inquiries can be directed to the corresponding author.
